# Intraductal Papillary Mucinous Neoplasm of the Bile Duct (IPMN-B): A Rare and Diagnostically Challenging Entity

**DOI:** 10.5334/jbsr.4141

**Published:** 2026-02-02

**Authors:** Jarno De Craemer, Louke Delrue, Koenraad J. Mortele

**Affiliations:** 1 Department of Radiology, Ghent University Hospital, Ghent, Belgium

**Keywords:** Intraductal papillary neoplasm of the bile duct, cystic liver lesion, biliary dilatation

## Abstract

*Teaching point:* Albeit rare, IPMN-B should be considered and excluded when encountering a large hepatic cystic lesion, especially in the setting of associated biliary dilatation, because in contrast to 95% of other hepatic cystic lesions, it has significant risk of harboring invasive carcinoma and therefore mandates early accurate diagnosis and complete surgical resection.

## Case History

**Figure 1:**
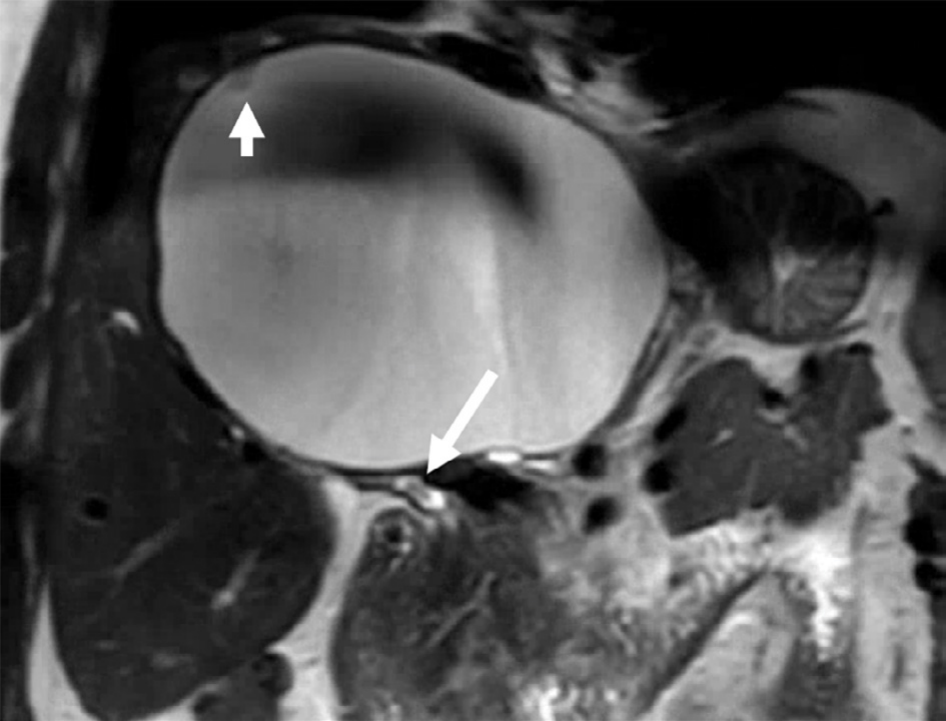
Coronal T2-weighted MR image shows a large cystic hepatic lesion with a small papillary projection (short arrow), peri-lesional intrahepatic bile duct dilatation, and a small filling defect in the extrahepatic bile duct (long arrow).

A 58-year-old Iranian male with long-standing vague abdominal symptoms and a large (presumed simple) hepatic cyst, for which he received several fenestrations and marsupialization at an outside institution, was referred because of increasing pain, cholestasis, and itchiness, and the possible diagnosis, on follow-up MRI, of a biliary cystadenoma and filling defect in the extrahepatic bile duct (Figure 1).

**Figure 2:**
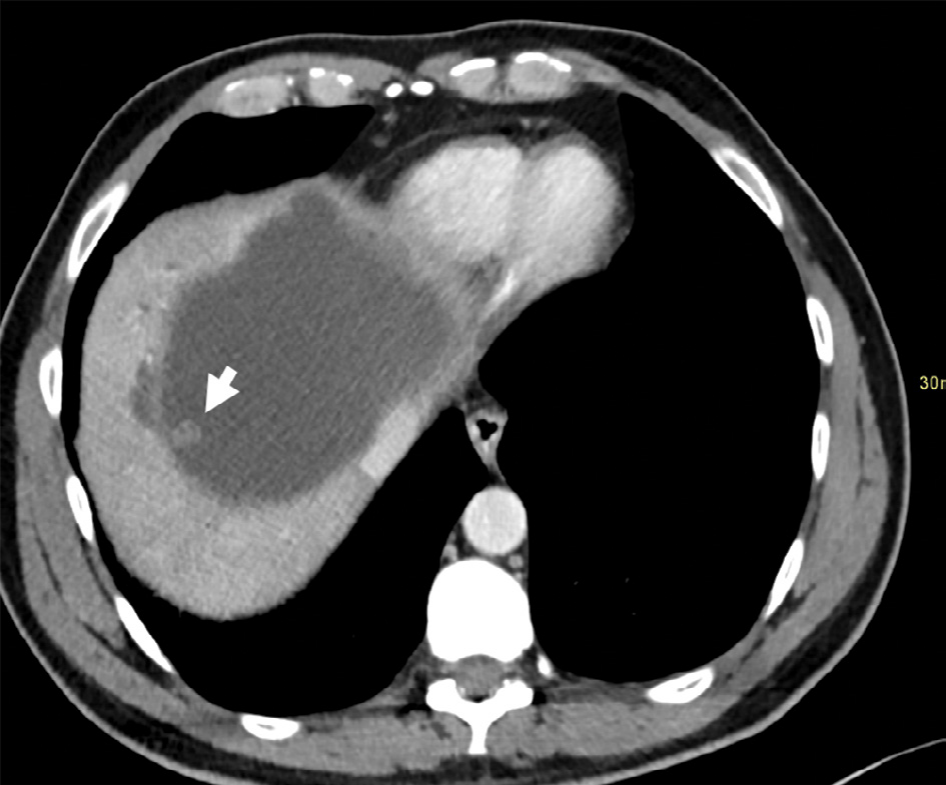
Axial contrast-enhanced MDCT image demonstrating the large cystic hepatic mass with papillary projection and bile duct dilatation.

**Figure 3:**
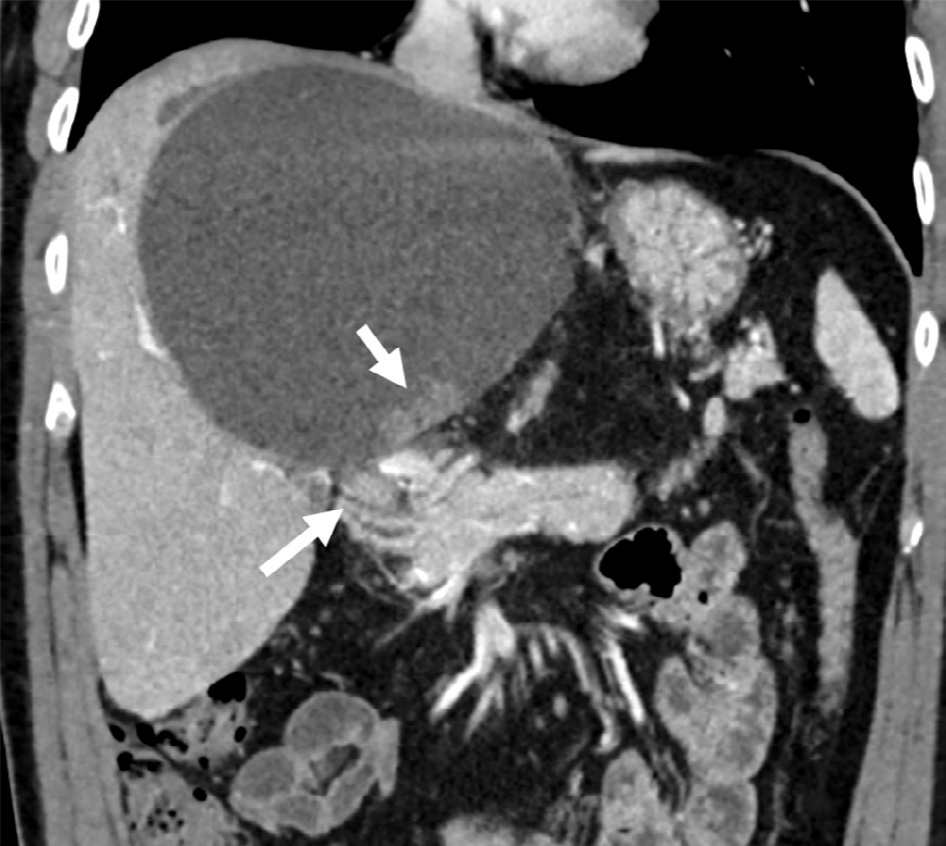
Coronal contrast-enhanced MDCT image demonstrating a new papillary projection (short arrow) and a grown soft tissue mass in the common hepatic duct (long arrow).

Repeat MDCT six months later (Figures 2 and 3) demonstrated a centrally located, large cystic lesion with few mural-based papillary projections. Associated intrahepatic bile duct dilatation was present, as also an oval shaped soft tissue mass in the common hepatic duct. Histopathological analysis of the partially resected cyst wall revealed IPMN-B with high-grade dysplasia.

## Comments

IPMN-B, a precursor lesion of cholangiocarcinoma, was defined in 2010 by the WHO as a mucin-producing papillary or villous neoplasm occurring in the bile ducts or peribiliary glands [1]. In contrast with its pancreatic counterpart IPMN-P, it is associated with known risk factors, such as hepatolithiasis, Clonorchis infection, PSC, etc., explaining its higher incidence in Far East countries. Contrary to biliary cystadenoma (also known as mucinous cystic neoplasm [MCN] of the liver) that nearly exclusively occurs in middle-aged female patients, IPMN-B is more common in older male patients (median age: 60–66; male-to-female ratio: 2:1–3:2).

The four described imaging phenotypes of IPMN-B depend on the size and morphology of the intraductal mass, the amount of mucin production, and tumor location. The ‘cystic’ subtype, with or without associated bile duct dilation, can be misdiagnosed, like in our case, as other cystic hepatic lesions including simple hepatic cyst (no mural nodules, rarely bile duct dilation), MCN (in women, rarely bile duct dilation, thick capsule common), and localized Caroli disease (intrahepatic only). Multiple lesions, as seen in our case, are described in up to 50% of cases, and may be located intrahepatic (most common) or extrahepatic. When present, the multifocality can be a very helpful clue to the correct diagnosis. Because the incidence of invasive carcinoma in IPMN-B (72%) is much higher than in IPMN-P (21%), early diagnosis and accurate treatment are of utmost importance.
